# Does trait anxiety influence effects of oxytocin on eye-blink startle reactivity? A randomized, double-blind, placebo-controlled crossover study

**DOI:** 10.1371/journal.pone.0190809

**Published:** 2018-01-04

**Authors:** Sonja Schumacher, Misari Oe, Frank H. Wilhelm, Michael Rufer, Markus Heinrichs, Steffi Weidt, Hanspeter Moergeli, Chantal Martin-Soelch

**Affiliations:** 1 Department of Psychiatry and Psychotherapy, University Hospital Zurich, University of Zurich, Zurich, Switzerland; 2 Department of Neuropsychiatry, Kurume University School of Medicine, Kurume, Japan; 3 Division of Clinical Psychology, Psychotherapy, and Health Psychology, Department of Psychology, University of Salzburg, Salzburg, Austria; 4 Department of Psychiatry, Psychotherapy and Psychosomatics, Psychiatric Hospital, University of Zurich, Zurich, Switzerland; 5 Department of Psychology, Laboratory for Biological and Personality Psychology, University of Freiburg, Freiburg, Germany; 6 Freiburg Brain Imaging Center, University Medical Center, University of Freiburg, Freiburg, Germany; 7 Division of Clinical and Health Psychology, Department of Psychology, University of Fribourg, Fribourg, Switzerland; Leiden University, NETHERLANDS

## Abstract

**Background:**

Previous research has demonstrated that the neuropeptide oxytocin modulates social behaviors and reduces anxiety. However, effects of oxytocin on startle reactivity, a well-validated measure of defense system activation related to fear and anxiety, have been inconsistent. Here we investigated the influence of oxytocin on startle reactivity with particular focus on the role of trait anxiety.

**Methods:**

Forty-four healthy male participants attended two experimental sessions. They received intranasal oxytocin (24 IU) in one session and placebo in the other. Startle probes were presented in combination with pictures of social and non-social content. Eye-blink startle magnitude was measured by electromyography over the musculus orbicularis oculi in response to 95 dB noise bursts. Participants were assigned to groups of high vs. low trait anxiety based on their scores on the trait form of the Spielberger State-Trait Anxiety Inventory (STAI).

**Results:**

A significant interaction effect of oxytocin with STAI confirmed that trait anxiety moderated the effect of oxytocin on startle reactivity. Post-hoc tests indicated that for participants with elevated trait anxiety, oxytocin increased startle magnitude, particularly when watching non-social pictures, while this was not the case for participants with low trait anxiety.

**Conclusion:**

Results indicate that effects of oxytocin on defense system activation depend on individual differences in trait anxiety. Trait anxiety may be an important moderator variable that should be considered in human studies on oxytocin effects.

## Introduction

The effect of the neuropeptide oxytocin on social behavior, anxiety, and stress reactivity has received increased attention in the last decade [1, 2]. Oxytocin may play a key role in the recognition of emotional facial expressions [3–6] and in related memory [7], interpersonal trust and cooperation [8], and attachment [9] in humans. Oxytocin also reduces behavioral and endocrine responses to social stress and mediates stress-protective effects of social support [10]. Moreover, recent studies suggest that the effect of oxytocin is dependent on social context [11]. Oxytocin might enhance the early and rapid conceptual detection of affect from social stimuli and might improve the accurate appraisal of affect from socially relevant information [12]. Animal and human research has begun to investigate the effects of oxytocin on the startle response, an important measure of defense system activation related to fear and anxiety [13].

The startle response is conceptualized as an involuntary reaction to an unexpected sudden intense stimulus that contracts several muscles in order to protect the body from harm [14]. Startle reactivity can be measured using the eye-blink reflex, which is part of the whole-body startle reaction and can easily be elicited by loud acoustic stimuli [15]. High startle reactivity is characterized by high eye-blink amplitudes. The startle response is highly context-dependent and can be modulated by many factors. In fear-potentiated startle, the startle response has significantly greater amplitude because the aversive motivational system, including the amygdala, is activated by a fear state [13, 16–18]. Thus, the startle response is a reliable index for fear in animal and human fear conditioning [19] and a valuable indicator for the analysis of neural systems associated with emotional activation, especially fear and anxiety, and emotion regulation [16].

The investigation of the effects of oxytocin on the startle response has led to contradictory results. Results of animal studies include increased startle response after oxytocin administration [20], no effects of oxytocin [21, 22], and reduced startle response after oxytocin administration [23]. In humans, a recent placebo-controlled double-blind study showed that the eye-blink startle magnitude was significantly larger in the oxytocin group than in the placebo group during the viewing of negative, but not neutral or positive pictures [24]. On the other hand, another study reported oxytocin to attenuate the startle reflex during the viewing of both emotional and neutral pictures, irrespective of picture valence [25]. Unfortunately, these two studies did not investigate social vs. non-social content of pictures, which might be an additional important factor as social context may be particularly prone to influence oxytocin effects [1].

Endogenous oxytocin has been shown to reduce anxiety and stress in the presence of fear-evoking stimuli [26, 27]. Therefore, it appears justified to assume that the application of exogenous oxytocin might lower levels of anxiety specifically in high trait anxious individuals. Consistent with this assumption, a recent study evaluating responses to speech stress showed that oxytocin reduced negative cognitive self-appraisals only in high trait anxious individuals [28]. On the other hand, and not consistent with this assumption, a recent animal study found that oxytocin reduced startle responses in the context of a fear experiment only in rats with overall low pre-fear startle responding[29], which may be considered a measure of trait anxiety in animals. In sum, the evidence on oxytocin affecting startle responding is quite inconclusive while trait anxiety appears to be an important moderator.

The aim of the present study was to shed more light on these initial and somewhat contradictory findings and investigate the relevance of individual differences in trait anxiety for influencing the effects of oxytocin on human defense system activation. We thus investigated the startle response during picture viewing, with a specific focus on the differences between social and non-social content of pictures. We tentatively expected to find an oxytocin-related reduction of startle responses particularly in high trait anxious participants. Based on the importance of oxytocin particularly in the context of social stimuli, we expected such an effect to be most pronounced with social compared to non-social stimuli.

## Methods

### Participants

Forty-four healthy non-smoking men were recruited through advertisements on pinboards and homepages. Exclusion criteria were neurological or psychiatric disorders, liver, kidney or heart diseases, allergy to preserving agents, use of medication including hormonal and herbal medication during the 4 weeks preceding the study, participation in other clinical trials during the 4 weeks preceding or during the study, impaired hearing (threshold over 30 dB), impaired cognitive abilities (IQ known to be below 70) and insufficient command of German. Physical and mental health was assessed by a structured screening interview (short medical history assessment) and by the German version [30] of the Mini-International Neuropsychiatric Interview (M.I.N.I.), a reliable and valid diagnostic structured interview [31, 32]. For sample information on age and trait anxiety, see Table 1. Participants were instructed not to consume alcohol or caffeine and not to exercise 24 hours before each session, and not to eat or drink anything besides water two hours prior to the assessments. Seven participants took part in only one of two sessions (see Table 1). Participants were thoroughly informed about the procedures and gave written informed consent according to the Declaration of Helsinki before participating. This study was approved by the local ethics committee and by the Swiss Regulatory Institute for drug trials (Swissmedic) and is registered as clinical trial at ClinicalTrials.gov, trial number NCT01066299. Monitoring of the study was performed by the Clinical Trial Center at the University Hospital Zurich.

**Table 1:** Sample description.

	**N**	**Range**	**Mean**	**SD**	**Median**					
**Both sessions**	37									
**Placebo session only**	4									
**Oxytocin session only**	3									
**Age in years**	44	18–51	27.2	8.6						
**STAI trait scores**	44	23–56	33.6	6.2	32.5					
** **	**Low trait anxiety**					**High trait anxiety**				
** **	**N**	**Range**	**Mean**	**SD**	** **	**N**	**Range**	**Mean**	**SD**	**p**
**Age in years**	22	19–43	26.5	7.9		22	18–51	28.1	9.8	0.6
**STAI trait scores**	22	23–32	29.0	2.4		22	33–56	37.6	5.1	<0.001

STAI = State-Trait Anxiety Inventory

### Procedure

The study took place at the psychophysiology laboratory at the Department of Psychiatry and Psychotherapy, University Hospital Zurich, Switzerland. We used a double-blind placebo controlled crossover design. Assessments were made on three visits. During the first visit participants were screened for inclusion and exclusion criteria and received a URL website link to fill in the German version of the trait form of the Spielberger State-Trait Anxiety Inventory [33] (STAI) online. Psychophysiological assessments took place during the second and third visits, which were separated by one to four weeks (M = 14.1 days, SD = 6.9 days). One week after the last visit participants were called for a follow-up check of their health status. Psychophysiological assessments took place at 2:00 p.m. in order to account for diurnal variation of oxytocin [10] and were structured as follows: How to use sprays was introduced to the participants before use. We used a written instruction, including a posture (light bow position), a depth of insertion (ca.1cm). Priming the sprays was conducted by the experimenters and confirmed its function before use. The experimenters did not observe any leakage. The experimenters observed the participants carefully. In addition, the study was conducted under the monitoring of the Center for Clinical Trial at the University Hospital. Therefore the standardized administration of the drug was performed under the strict supervision of our monitor.

First, 24 IU of an intranasal spray was administered (three puffs of four IU per nostril). Either oxytocin (Syntocinon, Novartis, Basel, Switzerland) or placebo (containing all ingredients except for the neuropeptide) was given at the first session and the other at the second session. The order was randomized across participants. After administration of the spray participants were left alone to read magazines for about 20 minutes. Then, sensors were attached while participants sat in a comfortable chair and they were subsequently asked to rest quietly for seven minutes in order to facilitate adaptation to the laboratory setting. Forty-five minutes after spray application, participants were given headphones and told that the session would start.

A background noise of 70dB was running throughout the session to mask any environmental sounds. Startle probes were presented as bursts of white noise (95 dB, 40 ms duration, sudden onset). Thirty-eight pictures from the International Affective Picture System (IAPS) [34] were shown for six seconds each with inter-trial intervals of 15–20 s (M = 17 s). The first two trials were not included in the analysis. These two habituation trials each consisted of a neutral picture and a startle probe. During the presentation of 24 of the remaining 36 pictures a startle probe was given 3.5 s, 4.0 s or 4.5 s after picture onset. Time points for the startle probes were varied to make them less predictable. Another six startle probes were given between pictures and 12 pictures were shown without a startle probe. The half of the pictures was of social (showing people) and the other half of non-social content. Participants were assigned to one of the two possible orders of set A and B, using the research randomizer (www.randomizer.org). We selected pictures with mild arousal scores between 3 and 5 from IAPS, because we planned to focus on the difference between social and non-social picture contents, not to focus on the affective stimulus. Each set consisted of 12 neutral, 12 positive and 12 negative pictures. A description of the picture sets is presented in Table 2. Task presentation was done via E-Prime 2.0 Professional (Psychology Software Tools Inc., Pittsburgh, PA., USA). Pictures were shown on a 19” computer screen and acoustic stimuli were presented binaurally via a Sony STR-DE197 amplifier and Novitronic sealed headphones. Noise volume level was calibrated using a Voltcraft SL-100 sound-level measuring device.

**Table 2:** Description of the two picture sets.

**Set A Social**					
**IAPS no.**	**Description**	**IAPS no.**	**Description**	**IAPS no.**	**Description**
2435	Mother & son	2091	Kids with kittens	2110	AngryFace
2487	Musician	2154	Father & son	2750	Bum
2579	Bakers	2311	Mother with daughter	2799	Funeral
3550.2	Coach	2360	Family	6311	Distressed woman
7496	People in street	2530	Couple	9220	Couple at cemetery
9171	Fisher	5831	Father & child at beach	9342	Men in polluted area
**Set A Non-social**					
**IAPS no.**	**Description**	**IAPS no.**	**Description**	**IAPS no.**	**Description**
5395	Ship	5200	Flowers	9000	Cemetery
5532	Mushrooms	5551	Clouds	9090	Exhaust
7037	Trains	5611	Mountains	9301	Toilet
7042	Barbells	5780	River	9373	Garbage
7095	Headlight	5814	Palm beach	9470	Destroyed house
7211	Clock	5910	Fireworks	9830	Cigarette stubs
**Set B Social**					
**IAPS no.**	**Description**	**IAPS no.**	**Description**	**IAPS no.**	**Description**
2305	Woman	2332	Infant with puppy	2053	Premature infant
2394	Medical worker	2339	Soccer player & kids	2141	Grieving woman
2515	Harvest	2373	Mariachi band	2399	Woman with headache
2593	Men in restaurant	2550	Old couple	3301	Injured child
2595	Women	5836	Couple at beach	9415	Handicapped
2635	Cowboy	8497	Roller coaster ride	9584	Dental exam
**Set B Non-social**					
**IAPS no.**	**Description**	**IAPS no.**	**Description**	**IAPS no.**	**Description**
7484	Grilled fish	5300	Galaxy	9320	Vomit
7560	Freeway	5594	Sky	9471	Burnt building
7920	Mud	5600	Mountains	9101	Cocaine
7242	Building	5631	Mountains	9290	Garbage
7058	Dice	5779	Courtyard	9340	Garbage
7057	Coffee cup	7545	Ocean view	9280	Smoke

We have no severe adverse events throughout our study, but there were eight cases with mild adverse events (e.g. exhausted, cold, nose bleeding); three were placebo and 5 were oxytocin. There were no differences in the adverse events reported between the two sprays (p = 0.48).

### Physiological measurement and data reduction

Recording of the physiological signals was performed using a BIOPAC MP150 system (Biopac Systems, Inc, Goleta, CA). Facial electromyographic (EMG) activity from the left musculus orbicularis oculi was recorded using Ag/AgCl electrodes filled with electrolyte gel. EMG was sampled at a 1000Hz rate. Autonomic Nervous System Laboratory 2.51 (ANSLAB; Wilhelm, F. H. & Peyk, P., 2005; available at the SPR Software Repository: http://www.sprweb.org) was used to filter raw data and extract startle responses. The startle EMG was 50Hz notch filtered, rectified and startle eye-blink magnitude (baseline corrected peak amplitude for response trials) was computed. The 50ms before the startle probe onset were used as baseline. The 100ms startle response window started 20ms after probe onset. Due to insufficient data quality, 3.3% of trials had to be excluded from analysis. To reduce inter-individual variance all startle eye-blink magnitudes were t-transformed within subjects and t-scores are reported.

### Data analysis

Statistical analyses were performed using IBM SPSS Statistics 23 (SPSS Inc., Chicago, Ill, USA). We used a linear mixed model design to compare conditions. Trait anxiety (STAI trait scores) was used as a covariate. The model for eye-blink magnitude during picture viewing contained Substance (oxytocin or placebo), Picture Content (social or non-social), STAI trait, as well as all possible interactions as fixed effects. After this analysis, to visualize and to decompose the significant interaction effects, STAI trait scores were median split and build two groups of participants with low vs. high trait anxiety scores (see Table 1). Bonferroni corrected pairwise comparisons based on the estimated marginal means were used as post-hoc tests.

## Results

**Fig 1:**
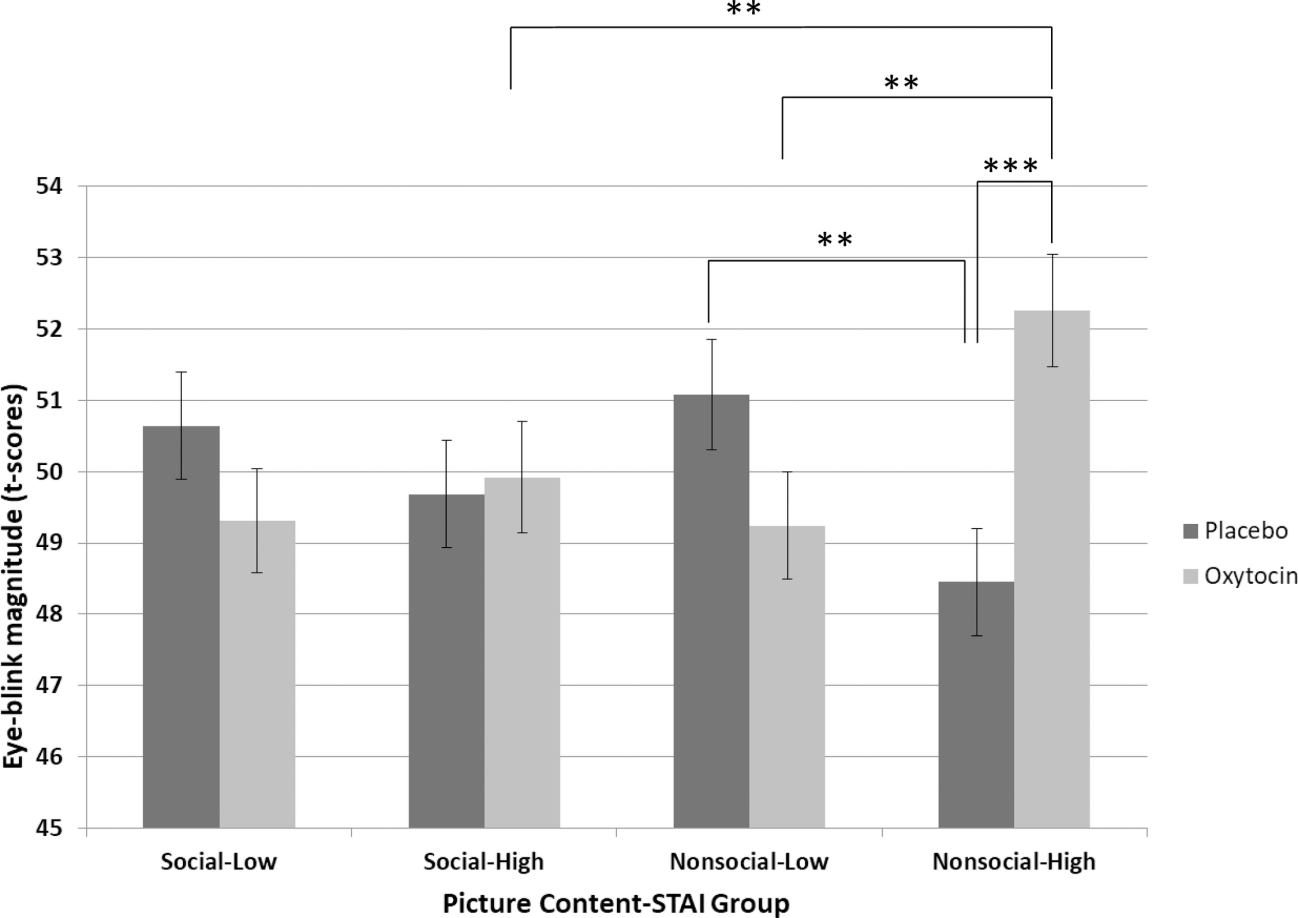
Estimated marginal means +/- 1 standard error for eye-blink magnitude (t-scores). Substance (placebo, oxytocin) x Picture Content (social, non-social) x STAI Group (low trait anxiety, high trait anxiety) Interaction effect, F (1, 1700) = 6.13, p = 0.013. *p<0.05, ** p < 0.01, *** p < 0.001.

There were no main effects of Substance (p = 0.769), Picture Content (p = 0.445), or STAI trait (p = 0.992). There was a significant interaction effect of Substance x STAI trait (F (1, 194) = 6.25, p = 0.013), and there was a trend interaction effect of Substance x Picture Content (F (1, 1667) = 29.4, p = 0.086); the significant Substance x STAI trait interaction effect was further qualified by a significant threefold interaction effect of Substance x Picture Content x STAI trait (F (1, 1700) = 6.13, p = 0.013). The analysis using STAI trait Groups instead of STAI trait scores yielded the same non-significant main effects and the same significant interaction effects. Decomposition of these interaction effects showed that significant differences were observed between oxytocin and placebo in individuals with higher STAI trait scores when watching nonsocial pictures (M_diff_ = 3.81, SE_diff_ = 1.07, 95% CI: 1.70 to 5.91, P < 0.001); between higher and lower STAI trait Group in nonsocial pictures with oxytocin (M_diff_ = 3.02, SE_diff_ = 1.09, 95% CI: 0.88 to 5.16, P = 0.006) and with placebo (M_diff_ = 2.63, SE_diff_ = 1.08, 95% CI: 0.51 to 4.75, p = 0.015); and between social and non-social pictures in higher STAI trait Group with oxytocin (M_diff_ = 2.34, SE_diff_ = 0.90, 95% CI: 0.59 to 4.10, P = 0.009) (Fig 1).

## Discussion

The main aim of this study was to investigate the influence of oxytocin on the startle reflex and how trait anxiety modulates this relationship. As expected, our results point to a modulatory role of trait anxiety on oxytocin effects on eye-blink startle during picture viewing. Specifically, participants with higher STAI trait scores showed stronger startle responses under oxytocin than under placebo when viewing pictures of non-social content. The startle reflex is a measure of defense system activation and defensive motivation [17]. Our results therefore suggest that for individuals with higher trait anxiety oxytocin increases defensive responding in a non-social context. These results complement those found by Striepens et al. (2012)[24] in contradicting an unspecific anxiety reducing effect of oxytocin. The differential effects of oxytocin on the startle reflex specific to high trait anxiety suggest that there are relevant interactions between individual trait characteristics and oxytocin. Anxiety reducing effects of oxytocin might be specific to certain personality traits in individuals under specific social-affective context conditions. However, the direction of the influence of trait anxiety was contrary to what we had expected. As Alvares et al. (2012) [28] had found stress-reducing effects of oxytocin in high trait-anxious individuals, we expected a startle-reducing effect of oxytocin in high trait-anxious participants. However, our data demonstrated stronger startle responses under oxytocin compared to placebo for high trait anxiety. A recent study found anxiety-enhancing effects of oxytocin in response to unpredictable threat [35]. In our study, startle-probe bursts were not cued and occurred at different time points within the picture presentation. Unpredictability might therefore in part explain the discrepancy between our results and those of Alvares et al. (2012) [28], where the stressor was continuous and therefore predictable.

One caveat of our results is the finding of contradictory results on the effect of oxytocin in social contexts. While a large empirical evidence supports a specific role of oxytocin on social settings, what gave rise to several socially-oriented accounts of oxytocin’s effects [36, 37] (for instance the prosocial theory or the social salience hypothesis of oxytocin), our results indicate an enhanced effect of oxytocin on the startle reflex in association with the presentation of the non-social pictures. This might be partly explained by the choice of our non-social stimuli. Contrary to previous studies (for instance, Striepens et al., 2012), we used non-social pictures from different contexts, while these authors chose their non-social pictures from one specific context (household and kitchen). It is therefore possible that this context variability influenced our results. In addition, we presented social and non-social stimuli for all three valence conditions (neutral, positive and negative), while previous studies on the effect of oxytocin on startle used only neutral non-social pictures. Finally, very few studies investigated the effect of oxytocin on the modulation of startle, and the research supporting the prosocial effects of oxytocin use different types of social stimulation (only pictures, or faces for instance). Our results indicate therefore that the choice of the social and non-social stimuli might influence the observed effects of oxytocin. This is also in line with the current reflections on the replicability of the social and behavioral effects of oxytocin.

There are several limitations to this study. First, since the level of high trait anxiety in our healthy male sample was still within a subclinical range, our results cannot necessarily be generalized to individuals with mental disorders associated with severe anxiety. Second, it also should be kept in mind that static pictures with social content are only a relatively mild social stimulus. More naturalistic social stimuli [38] might have produced different results. Third, we only investigated men in order to exclude endocrine influences of the female menstrual cycle. It is a major limitation that sex differences could not be tested in the current study. Fourth, several factors including anatomy of the nose and airways, nasal cavity environment, the nasal spray formulation, the bottle design, using standardized instructions across experiments [39], or pharmacokinetics and dose-dependncy on human brain reactivity [40] could influence the variability of response to oxytocin nasal spray. Fifth, it is not possible in our study to control for the effect of probe latency, and of the variation of interstimulus intervals on startle, because we did not keep data about randomization of the probe latency.

In conclusion, our results show that oxytocin affects the human defense system activation in complex ways. As the effect of oxytocin on the startle response depended on trait anxiety, future studies may benefit from taking this important individual-difference variable into account. A neglect of trait anxiety in most oxytocin studies on startle reactivity may be partially responsible for previous contradictory results. Further studies in clinical populations are needed to understand these effects in the framework of anxiety and stress related disorders and their relationship to oxytocin interventions for these conditions.

## Supporting information

S1 Dataset The dataset of the study. (SAV)
